# Real-World Analysis of Remote Electrical Neuromodulation (REN) for the Acute Treatment of Migraine

**DOI:** 10.3389/fpain.2021.753736

**Published:** 2022-01-18

**Authors:** Jessica Ailani, Liron Rabany, Shira Tamir, Alon Ironi, Amaal Starling

**Affiliations:** ^1^Georgetown University Hospital, Headache Center, Washington, DC, United States; ^2^Theranica Bio-Electronics LTD., Netanya, Israel; ^3^Mayo Clinic, Department of Neurology, Scottsdale, AZ, United States

**Keywords:** migraine, headache, neuromodulation, real world, treatment, REN, remote electric neuromodulation, medication

## Abstract

**Introduction**: Migraine is a chronic neurological disease that is the primary cause of years lived with disability in people under the age of 50. Remote electrical neuromodulation (REN) is a novel drug-free acute treatment of migraine, that is FDA cleared for episodic and chronic migraine. As a prescribed digital therapeutic, REN enables large-scale post-marketing research, thus providing real-world information on the use of the intervention in a wide range of populations, environments, and situations.

**Methods**: The REN device (®Nerivio) includes a secured, personal migraine diary, which patients can use to record their symptoms before treatment and 2 h post-treatment. Real-world data on REN treatments were collected via the app from patients across the United States who used Nerivio between October 1st, 2019, and May 24th, 2021. Data analysis focused on four metrics: 1. Per-treatment patterns of REN use as a standalone treatment vs. in combination with medications. 2. Per-user intra-individual efficacy across multiple treatments. 3. Distribution of treatment intensity among users (the electroceutical equivalent to treatment dose). 4. Prevalence and severity of adverse events.

**Results**: 1. Out of 23,151 treatments, in 66.5% of treatments REN was used as a standalone treatment, in 12.9% it was followed by over-the-counter medications, and in 20.6% followed by prescription medications. 2. Out of 2,514 patients, response in at least 50% of treatments was achieved in 66.5% of cases for pain relief, and in 22.6% for pain freedom. 3. Out of 117,583 treatments, in 80% of cases intensity levels were between 18 and 55% of the stimulator's range. The mean intensity was 34.3% of the stimulator's output (±16.6%). 4. Out of 12,368 users (121,947 treatments), there were 59 users (0.48%) who reported device related adverse events, 56 (0.45%) of which were mild, three (0.03%) were moderate, and none were severe.

**Conclusions**: The current analysis of real-world clinical data indicates that REN provides an efficacious, stable, and safe treatment option for acute treatment of migraine in real-world settings, both as a standalone replacement of pharmaceuticals, as well as an adjunct to medications.

## Introduction

Migraine is one of the most prevalent and disabling diseases worldwide, affecting ~12% of the global population (1). It is characterized by headache attacks that are recurrent and disabling, and often associated with nausea, vomiting, photophobia, and phonophobia (2). Migraine attacks are often treated with acute pharmacological care that includes over-the-counter (OTC) analgesics such as acetaminophen or aspirin (sometimes combined with caffeine), non-steroidal anti-inflammatory drugs (NSAIDs), or with specific migraine treatments such as triptans, ergots, gepants, and lasmiditan (3). However, these treatments may be contraindicated, poorly tolerated, ineffective, and if used frequently, some may lead to medication overuse headache (MOH) (4–7).

An alternative treatment for migraine is non-invasive neuromodulation. Remote electrical neuromodulation (REN) is a novel drug-free acute treatment for migraine (8–10), which activates one of the body's endogenic pain management mechanism- conditioned pain modulation (CPM). CPM is a descending endogenous analgesic mechanism in which a sub-threshold stimulation inhibits pain in remote body regions (11). The REN device is an FDA-cleared wearable, wireless, battery-operated stimulation unit (®Nerivio), controlled by a smartphone application. The device is applied for 45 min to the lateral upper arm.

Randomized controlled clinical studies have demonstrated that REN is safe and effective for the acute treatment of migraine in adults with episodic migraine (8–10, 12–14), adults with chronic migraine (15), and adolescents with migraine (16). A comparison of the efficacy of REN to that of acute migraine medications indicated that REN has non-inferior efficacy compared to the tested acute migraine therapies (10). Other neuromodulation techniques were also found effective for the acute treatment of migraine, including transcutaneous electrical nerve stimulation [TENS; (17)], repetitive transcranial magnetic stimulation [rTMS; (18)] and transcranial direct current stimulation [tDCS; conventional and high definition (19)]. However, a recent systematic review and meta-analysis found that REN was the only migraine neuromodulation intervention for which there is sufficient high-quality research, and thus the only one for which efficacy was well-established (20).

Furthermore, REN is recommended by the recent American Headache Society Consensus Statement (21) as an adjunct to the existing treatment plan for patients with an inadequate response to a migraine-specific acute medication, as well as those with frequent attacks who may be at risk of developing medication-overuse headache and/or chronic migraine due to overuse of acute medication.

Post-marketing studies (also termed real-world evidence analyses, or phase IV studies) assess the effectiveness and usability of novel treatments, in larger and more diverse populations, and in various real-world environments and situations. As such they are essential for the true evaluation of the effects of any intervention, and are recommended by the International Headache Society (IHS) as part of the guidelines for clinical trials with neuromodulation devices for the treatment of migraine (22).

As a prescribed digital therapeutic (i.e., electroceutical), the REN device enables prospective (real-time) collection of electronic patient-reported outcomes in real-world use, in a large group of users. We investigated clinical benefits and pattern of use in real-world users, focusing on four objectives: The first objective was to evaluate the prevalence of REN use as a standalone acute therapy vs. in combination with migraine medications, and the efficacy of standalone and combination treatments. The second objective was to measure the efficacy of REN across multiple treatments. The third objective was to explore the distribution of treatment intensity among REN users (intensity refers to the output of the stimulator, as determined by the patient via the application. It thus can be considered as an equivalent to the dose of the treatment). Lastly, the fourth objective was to quantify the prevalence and type of device-related adverse events (AEs), in order to provide information on real-world use safety.

Together, these four objectives provide a comprehensive evaluation of efficacy, drug-device interactions, dose stability, and safety, in a large real-world dataset of over 100,000 treatments.

## Materials and Methods

### The REN Device

The REN device has been described in detail previously (9). Briefly, REN is a wearable device applied to the upper arm and stimulates the ascending pain pathway using a modulated, symmetrical, biphasic, square pulse with a pulse width of 400 μs, modulated frequency of 100–120 Hz, and up to 40 mA output current which can be adjusted by the patient.

### Data Collection

As part of the sign-up process to the Nerivio app, all patients accepted the terms of use which specify that providing personal information is done of their own free will, and that their de-identified data may be used for research purposes. Users were not obligated to provide personal information and could treat without providing any feedback. The app includes a secured, personal migraine diary, which enables patients to record and track their migraines and other headaches. At the beginning of each treatment, and again 2 h after the start of treatment, patients are prompted to record their symptoms, including pain level (none, mild, moderate, severe), functional disability (“No limitation,” “Some limitation,” “Moderate limitation,” “Severe limitation”), and indication of which medications, if any, were taken within that 2-h time window.

### Dataset

Real-world data of REN treatments were collected from patients across the United States who used the REN device between October 1st, 2019 and May 24th, 2021.

“***Treatment***” was defined as a REN treatment of at least 20 min (the nominal duration is 45 min).

“***Evaluable treatment***” was defined as a ***treatment*** in which pain levels were reported at baseline and post-2 h.

Inclusion criteria for each of the different metrics were as follows:

1. REN-medication combinations: all evaluable treatments.
2. Efficacy across multiple treatments: all users that performed at least 2 evaluable treatments. In order to isolate the effect of REN treatments, this dataset considered only treatments where REN was used as a standalone treatment.
3. Treatment intensity: all treatments.
4. Safety: Safety was evaluated based on all reported adverse events (AEs) within the time period (these data were collected as reports to both the company or the FDA, not via the Nerivio app), and are reported along with the number of users and treatments of all REN sessions, regardless of duration.

### Outcome Measures

Respectively, these outcome measures were tested:

#### REN-Medication Combinations (Prevalence and Efficacy)

Medication intake outcomes were calculated based on the 2 h post-treatment report and comprised of the percentage of treatments in which no rescue medications were used, treatments in which OTC medications were taken, treatments in which triptans were taken, treatments in which other prescription medications were taken, and treatments in which medication intake status was not reported. OTC included acetaminophen, NSAIDs, and combinations of the two, with or without caffeine. Treatment in which there was no report regarding medications intake are presented to provide full information, but were not included in the analysis of this endpoint.

Efficacy outcomes were calculated per treatment based on the baseline and post-2 h reports and comprised of:

(i) pain relief (decrease in headache from moderate or severe at baseline to mild or no pain at post-2 h); (ii) pain-freedom (decrease in headache from mild, moderate, or severe at baseline to no pain at post-2 h); (iii) improvement in function (improvement of at least one grade between baseline and post-2 h, for treatments in which limitation was reported at baseline); and (iv) return to normal function (a report of no functional disability at 2 h, for treatments in which limitation was reported at baseline).

#### Efficacy Across Multiple Treatments

The proportion of individuals that achieved a response to treatment in at least 50% of their treatments was calculated for the four efficacy outcomes: (i) pain relief (ii) pain-freedom (iii) improvement in function; and (iv) return to normal function.

This analysis included only treatments where REN was used as a standalone treatment.

#### Treatment Intensity

The mean intensity of the stimulation was collected for all treatments with a duration of 20 min or longer, which were performed within the study's time window. The data is presented as a histogram, along with mean (±SD), median, quartile, and decile information (and along with similar data from clinical trials for REN).

#### Safety

All adverse events that were reported within the study's period were analyzed and the following information is provided: number of device-related AEs, percentage of the device-related AEs that were mild, moderate, and severe, and percentage of AEs that were serious vs. not serious.

## Results

The sample consists of users who have voluntarily chosen to report their symptoms and medication intake. As these data were collected from real-world use, users were not explicitly requested, let alone obliged, to report. The differences in sample sizes between the different analyses reflect the differences in the inclusion criteria for each analysis.

For demographic and clinical data of the patients included in each dataset (see Table 1).

**Table 1:** Sample sizes and demographics of patients in each one of the analyses.

**Analysis**	***N*, Subjects**	**Age, Mean ±SD**	**Gender (% female)**	**Prescribed by headache clinic/primary care**
Medication combinations	5,805	43.3 ± 14.2	87.8%	83.0%/17.0%
Efficacy across treatments	2,514	44.0 ± 14.3	86.8%	84.3%/15.7%
Treatment intensity	12,151	43.9 ± 14.6	85.5%	86.7%/13.3%
Safety	12,368	43.9 ± 14.6	85.1%	86.4%/13.6%

The vast majority of patients in all the analyses (>80%) were prescribed Nerivio by headache specialists, and the rest by primary care physicians. The average number of treatments per person was 9.68. Out of the 12,151 users who had valid treatments, 28.6% (*n* = 3,475) had only one or two treatments, 47.0% (*n* = 5,709) had 3–10 treatments, 24.4% (*n* = 2,967) had more than 10 treatments, and 2.4% (*n* = 291) had more than 50 treatments.

### REN-Medication Combinations

Data from 23,151 treatments (performed by 5,805 patients) were eligible for analysis.

In 822 treatments no medication report was available. Out of the 22,329 treatments for which a medication report was available: in 66.5% (*n* = 14,854) of the treatments REN was used as a standalone acute therapy, in 12.9% (*n* = 2,886) REN was used in combination with over-the-counter medications, in 11.2% (*n* = 2,497) REN was used in combination with triptans, and in 9.4% (*n* = 2,092) REN was used in combination with other prescription medications. Table 2 describes the response to treatment outcomes, per medication intake status.

**Table 2:** Response to treatment outcomes, per status of acute medication intake.

	**Endpoint (post-2 h)**							
	**Pain relief**		**Pain freedom**		**Functional disability improvement**		**Return to normal function**	
**Medication intake**	**% Treatments**	**n/N**	**% Treatments**	**n/N**	**% Treatments**	**n/N**	**% Treatments**	**n/N**
REN only (no meds)	55.6%	6,519/11,723	20.3%	3,021/14,854	51.2%	6,731/13,156	24.9%	3,272/13,156
REN & OTC	51.3%	1,226/2,391	15.5%	447/2,886	49.6%	1,305/2,630	19.7%	517/2,630
REN & Triptan	42.0%	859/2,045	12.3%	308/2,497	39.8%	911/2,288	13.0%	297/2,288
REN & Other Rx	38.5%	708/1,839	10.1%	212/2,092	44.7%	886/1,980	11.0%	218/1,980
No report of meds status	54.7%	363/664	18.2%	150/822	54.9%	353/643	22.7%	146/643

*n, number of treatments in which the endpoint was achieved; N, number of treatments for which data was available*.

### Efficacy Across Multiple Treatments

Data from 2,514 patients who used REN as a standalone treatment (12,735 treatments) were eligible for the analysis of this endpoint. Overall, 66.5% of the users achieved pain relief in at least 50% of their treatments, 22.6% of the users achieved pain freedom in at least 50% of their treatments, 61.3% of the users achieved reduction in functional disability in at least 50% of their treatments, and 29.8% of the users achieved return to normal function in at least 50% of their treatments. Table 3 presents the percentages of patients achieving response to treatment, per prescriber type (headache clinic/primary care), for each of the efficacy outcomes.

**Table 3:** Efficacy outcomes across multiple treatments, per prescriber affiliation.

	**Prescriber affiliation**			
	**Headache clinic**		**Primary care**	
**Endpoint (at least 50% of treatments, per person)**	**% Users**	**n/N Users**	**% Users**	**n/N Users**
Pain relief	64.4%	1,296/2,012	77.8%	284/365
Pain freedom	20.9%	442/2,119	32.2%	127/395
Improvement in function	60.2%	1,246/2,071	67.5%	258/382
Return to normal function	27.7%	573/2,071	41.6%	159/382

*n, number of users for which the endpoint was achieved; N, number of users for which data was available*.

Note that while data from 2,514 subjects were available for pain freedom status, the other outcome measures require that a severity criterion would be met to qualify for the analysis, and thus these outcome parameters have a slightly lower number of subjects (see methods section, i.e., “pain relief” is defined as a reduction from a pre-treatment severity level of moderate or severe to mild or none, meaning that individuals with a baseline severity of mild were not included in that specific outcome parameter).

### Treatment Intensity

**Figure 1:**
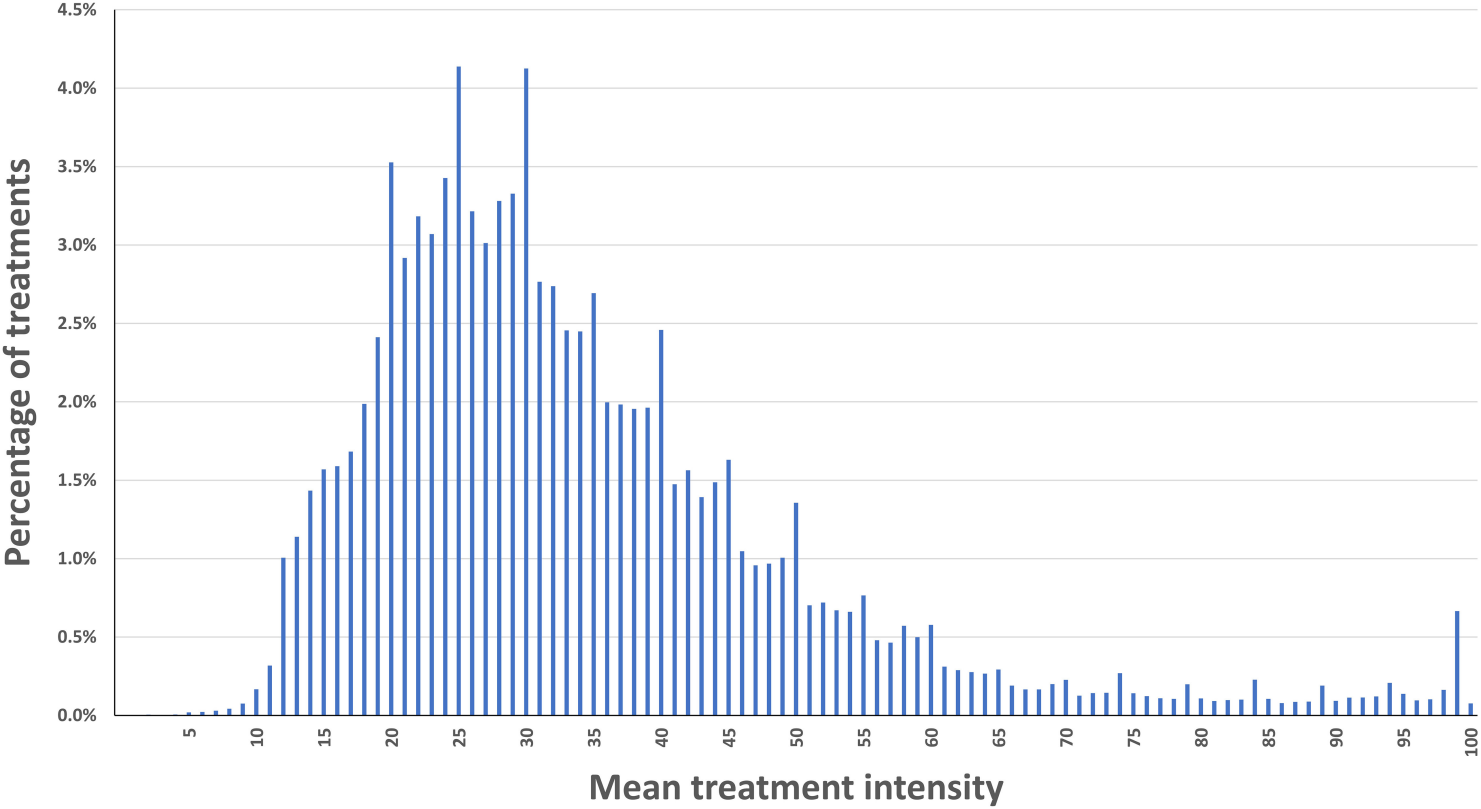
Distribution of mean treatment intensity.

One lakh seventeen thousand five hundred and eight-three treatments from 12,151 REN users were eligible for the analysis. The mean intensity was 34.3% of the maximal stimulator output (full output 40 mA), with an SD of 16.6%, and a median of 30%. Eighty percent of the users applied intensity levels between 18 and 54% of the stimulator's range (Decile one = 18, decile nine = 55). Figure 1 presents a histogram of the mean intensity distribution in the current dataset.

### Safety

The overall number of treatments in the analyzed time period was 121,947, conducted by 12,368 users. The total number of patients who reported device related AE reports in that period was 59 (0.48%), out of which 56 (0.45%) were mild, three (0.03%) were moderate, and none were severe. Local paresthesia in the area of the device AE, and a momentary painful increase in the stimulation intensity were the most common side effects. No severe AEs were reported.

## Discussion

Post-marketing surveillance studies involve systematic monitoring of interventions as they are used in real-life scenarios, as opposed to the controlled settings of pre-marketing trials, where study conditions are tightly controlled. As such, they provide essential information on the usability and effectiveness of novel treatments (23).

The current analysis demonstrated that REN provides an efficacious, stable, and reliable treatment option. The analysis focused on four key parameters. First, a large-scale analysis of over 23,000 treatments showed that in nearly 80% of treatments in which medication status was reported no prescription medication was taken within 2 h from treatment onset. Of which, in 66.5% of treatments no medication whatsoever was taken, and in 12.9% an OTC medication was taken. Given that 83% of the subjects in this analysis were patients of headache clinics, i.e., almost all of them were on prescription medications prior to being prescribed with REN (24), the use of prescribed medications while using REN—only 20.6% of the treatments—is very low. This suggests that treating with REN results in a substantial reduction in intake of prescribed pharmacological medications (and consequently may reduce the risk of developing medication overuse headache). The current data also support the willingness of patients to adopt a drug-free treatment.

In terms of REN's efficacy across multiple treatments (analyzed in treatments where no rescue medications were taken, to isolate the effect of REN), 66.5% of the users achieved pain relief in at least 50% of their treatments, and 22.6% achieved pain freedom in at least 50% of their treatments. Furthermore, 61.3% achieved improvement in functional disability in at least 50% of their treatments, and 29.8% achieved return to normal function in at least 50% of their treatments. These results align with findings from clinical trials examining REN (9, 15, 16). Further analysis of that group indicates that 84% of those patients were prescribed REN by headache specialists, representing a difficult to treat population for which OTC treatments typically do not suffice. Efficacy analysis of that difficult to treat sub-sample indicates that 64.4% achieved pain relief in at least 50% of their treatments, 20.9% achieved pain freedom in at least 50% of their treatments, 60.2% achieved improvement in functional disability in at least 50% of their treatments, and 27.7% achieved return to normal function in at least 50% of their treatments. The subgroup of 16% patients who were prescribed by primary care physicians had even better efficacy results, with 77.8% of users that achieved pain relief in at least 50% of their treatments, 32.2% achieved pain freedom in at least 50% of their treatments, 67.5% achieved improvement in functional disability in at least 50% of their treatments, and 41.6% achieved return to normal function in at least 50% of their treatments. Overall, these results indicate that the high efficacy demonstrated in controlled clinical trials is reproduced in real-world use, in large cohorts, with high rates of response among difficult to treat patients, and even more so in the general migraine population.

The results in the groups that took rescue medications within 2 h from treatment onset were slightly lower than those achieved in the group that used REN only, with a gradient from highest efficacy for the combination of REN and OTC, lower for the combination of REN and triptans, and lowest for REN and non-triptan migraine prescription medications. These results may stem from several reasons: a likely explanation is that patients with more severe migraines (i.e., patients taking non-triptan prescription medications, and to a lesser degree those who take triptans) are less responsive to any migraine treatment. Another possible explanation is that those who had sufficient improvement following REN did not need to take any additional medications, while those who were still experiencing some pain added a second treatment after a while, but that treatment may not have had sufficient time to reach its full effect by the time of the post-2 h pain report.

Our analysis of the stimulation intensity in over 100,000 treatments (performed by 12,151 users) during a period of 20 months indicates that the intensity parameters are similar to those measured in REN's controlled clinical studies [Mean ± SD in (9, 15, 16): 33.2 ± 15.5, 26.7 ± 12.5, 31.5 ± 14.5, respectively]. This similarity further ensures the usability of REN and the effective transition from clinical trials to real-world use. Additionally, while different patients use different intensity levels to reach their sub-painful sensation, 80% of the users apply intensity levels between 18 and 54% of the stimulator's range. Importantly, Nerivio's electrical stimulation intensity range is designed so that even the maximum possible intensity (“100%”) complies with the IEC 60601-1 and IEC 60601-2 safety guidelines.

Real-world analyses efficacy data are often collected via retrospective evaluations (questionnaires) which are limited by patients' recollection abilities, or collected via prospective naturalistic-designed clinical studies which may introduce selection bias. The mobile application of the REN device, which is connected to a dedicated HIPAA-compliant data server, allows patients to record their symptoms in real-time and provides the opportunity to collect prospective, real-time efficacy data similarly to controlled clinical studies, but in a real-world environment.

The neural mechanisms which facilitate the abortive effect of REN stimulation over migraine, have been discussed in detail elsewhere (9). Briefly, REN stimulates nociceptive nerve fibers in the upper arm to activate an endogenous descending pain inhibition mechanism termed Conditioned Pain Modulation [CPM; (11, 25, 26)]. An fMRI study (27) in healthy subjects found that CPM is associated with signal changes in brainstem regions, and that the magnitudes of these signal changes was correlated with the magnitude of CPM-induced analgesia. Another recent MRI study (28) found that higher resting connectivity between the periaqueductal central gray (PAG) and cortical pain processing regions, was associated with more efficient inhibitory CPM, and that higher resting connectivity between the PAG and cortical pain processing regions was associated with more efficient inhibitory CPM in healthy participants. Additionally, greater PAG connectivity to the rostral ventromedial medulla (RVM), was pain-inhibitory. These findings align with aberrations in brainstem regions (including the PAG and RVN) found in migraine patients during the interictal phase and throughout the migraine cycle [e.g., (29)], and could be part of the subserving mechanism of action. Relatedly, brainstem regions are also indirectly activated by other neuromodulation techniques for treatments for migraine such as TMS (30) and tDCS (31) targeted at cortical regions.

The current study has a few limitations. First, data were collected only from those patients who chose to use this intervention, which in some cases may imply that they were not satisfied with their previous therapies. Second, data were collected only from those patients who chose to use the app to report their symptoms. Nevertheless, real-world studies are essential for true evaluation of the effects of an intervention, as they test larger and more diverse populations over longer periods of time and are conducted in various real-world environments and situations. The use of a single parameter for intra-individual efficacy across multiple treatments removes the potential bias that could have been implied from users that treated many times (presumably because they experienced efficacy) over users who treated only very few times (perhaps because of lack of efficacy), since in this kind of analysis frequent users have the same weight as infrequent users. Lastly, as in all interventions, a certain degree of placebo-effect is to be expected. A previous double-blind randomized clinical trial of REN (23) indicated a placebo response at a rate of 38.8% for 2-h pain relief (vs. 66.7% change in the active treatment group). This information could serve as a point of reference, indicating that while a placebo effect no doubt exist, it is not likely to explain the current results.

## Conclusions

The current real-world clinical data confirm the findings of pre-marketing studies, namely that REN provides an efficacious, stable, and reliable treatment option for acute treatment of migraine in real world settings, both as a standalone replacement of pharmacological medications—the majority of use cases, or as an adjunct, in some cases.

## Data Availability Statement

The raw data supporting the conclusions of this article will be made available by the authors upon request, without undue reservation.

## Ethics Statement

Ethical review and approval was not required for the study on human participants in accordance with the local legislation and institutional requirements. Written informed consent from the participants' legal guardian/next of kin was not required to participate in this study in accordance with the national legislation and the institutional requirements.

## Author Contributions

LR drafted the manuscript, with the help of all other authors. LR and AI designed the analysis. ST and LR performed the analysis. JA and AS reviewed the analysis and provided important comments and changes to the analysis and the manuscript. All authors participated in the interpretation of the data and revising of the manuscript, contributed important intellectual content, and approved the final version of the manuscript.

## Funding

This study was funded by Theranica Bio-Electronics Ltd.

## Conflict of Interest

The authors declare that this study received funding from Theranica Bio-Electronics Ltd. The funder had the following involvement in the study: the funder designed and performed the analysis together with the other authors, and took part in the writing of the manuscript. AI, LR, and ST are employees of Theranica Bio-Electronics Ltd and hold stocks.

## Publisher's Note

All claims expressed in this article are solely those of the authors and do not necessarily represent those of their affiliated organizations, or those of the publisher, the editors and the reviewers. Any product that may be evaluated in this article, or claim that may be made by its manufacturer, is not guaranteed or endorsed by the publisher.

## Abbreviations

REN: remote electrical neuromodulation
OTC: over-the-counter (medications)
NSAID: non-steroidal anti-inflammatory drug
MOH: medication overuse headache
CPM: conditioned pain modulation
HIS: International Headache Society
AE: adverse events.
